# Editorial: Leishmaniasis and immunity: challenges, advances and future perspective

**DOI:** 10.3389/fimmu.2025.1666020

**Published:** 2025-08-06

**Authors:** Sarfaraz Ahmad Ejazi, Abhay Satoskar, Neetu Singh

**Affiliations:** ^1^ Fischell Department of Bioengineering, A. James Clark School of Engineering, University of Maryland, College Park, MD, United States; ^2^ The Ohio State University, Columbus, OH, United States; ^3^ The University of Texas MD Anderson Cancer Center, Houston, TX, United States

**Keywords:** leishmaniasis, host-parasite interactions, immune response, parasite survival, visceral leishmaniasis, cutaneous leishmaniasis

Leishmaniasis is still one of the most prevalent parasitic diseases in tropical and subtropical regions of the world (1). Despite sustained global efforts and collaborative initiatives over the past two decades, cutaneous leishmaniasis (CL) continues to account for hundreds of thousands of new cases annually (2). Meanwhile, visceral leishmaniasis (VL) persists as the second most fatal disease among parasitic diseases after malaria in these regions. Although over a century has passed since the discovery of pentavalent antimonials, the emergence of drug resistance in several endemic areas has significantly reduced its efficacy (3). Currently, most therapeutic options in use or under clinical evaluation for leishmaniasis are repurposed drugs, underscoring the need for new treatment strategies. Molecular tools have considerably improved the detection of *Leishmania* species and identification of asymptomatic infections, facilitated by point-of-care diagnostics in resource-limited field settings. However, the development of an effective and accessible vaccine remains elusive (4).

The biology of *Leishmania* infection is inherently complex, with different species causing distinct clinical manifestations influenced by multiple factors, most notably host-parasite interactions, which ultimately determine disease outcome (5). To survive within the hostile environment of host macrophages, the parasite has evolved various immune evasion strategies (6). Therefore, a deeper understanding of the dynamic interplay between parasite virulence mechanisms and host immune defenses, particularly in an epigenetic framework, is essential for developing effective interventions against the disease. Recent studies have emphasized the key role of host immunity in disease progression, treatment response, and potential vaccine development. Identifying these immunological determinants is crucial to advance our knowledge on *Leishmania* infection. This Research Topic brings together cutting-edge research addressing the multifaceted challenges of leishmaniasis, explores advances in immunological research and provides future perspectives. We hope the contributions compiled here not only update current knowledge on the topic but also inspire novel strategies to combat this enduring global health burden.

The Research Topic begins with a perspective article by Kumar et al., which offers a comprehensive overview of post kala-azar dermal leishmaniasis (PKDL), a cutaneous sequel of VL. The review highlights recent advances in diagnosis, treatment, and immunological understanding of PKDL. Importantly, the authors discuss the implications of PKDL in VL elimination programs, outlining key challenges and proposing strategic approaches to address them. Ihedioha et al. explored the host–pathogen interactions during *L. major* infection, focusing on how these dynamics influence disease progression. Their study examined the role of lipophosphoglycan (LPG), a key surface virulence glycoconjugate in platelet activation and the induction of Dickkopf-1 (DKK1), a modulator of proinflammatory responses. Utilizing *L. major* mutant strains lacking LPG synthesis, the authors demonstrated that LPG mediates platelet activation through TLR1/2 signaling and promotes the formation of leukocyte–platelet aggregates. Their findings suggest LPG-activated platelets contribute to Th2 immune polarization, shaping the host’s immune response against *Leishmania* infection.

Advances in leishmaniasis research have also pointed out the role of various factors, particularly the host’s genetic background, on disease susceptibility and protection. In this context, Junior et al. conducted a haplotype-based analysis using single nucleotide variants (SNVs) across a chromosomal region to identify genetic markers associated with clinical outcomes. Their results revealed that three human IL-13 gene haplotypes conferred protection against *L. guyanensis*-induced CL, whereas three others were associated with increased susceptibility to the disease. It is well established that many pathogens enhance their intracellular survival by manipulating host RNA interference (RNAi) pathways and their associated components. In a study, Moradimotlagh et al. explored the involvement of Argonaute (Ago) protein complexes in the pathogenesis of *L. donovani*. Their findings revealed that infection with *Leishmania* selectively upregulates Ago1 in host macrophages and that silencing Ago1 significantly reduced parasite survival. Furthermore, proteomic profiling identified several *Leishmania*-associated pathogenic proteins whose expression correlated with Ago1 levels in infected macrophages, suggesting a potential role in modulating host responses and promoting disease progression.

Bernardo et al. investigated how immunosuppressive therapies, including anti-TNF agents and methotrexate (MTX), affect the host immune response and the efficacy of anti-leishmanial therapy during *L. infantum* infection. Their study demonstrated that immunosuppressed mice exhibited impaired parasite clearance and a reduction in proinflammatory cytokine production following treatment. This diminished response was likely due to the host’s inability to mount a specific cellular immune defense against the parasite under immunosuppressive conditions. In an *in vivo* study, Devender et al. evaluated the immunogenic potential of the tuzin protein as a vaccine candidate against *L. donovani* infection. Mice immunized with tuzin showed a significant reduction in parasite burden and elevated levels of Th1-associated immune markers. Following parasite challenge, tuzin-immunized mice exhibited an increased IFN-γ/IL-10 ratio, indicating a protective Th1-biased immune response against *L. donovani*.

de Araujo et al. investigated the immune response of healthy volunteers exposed to the saliva of uninfected *Phlebotomus duboscqi*, a known vector of *L. major*. The study reported significant antigen-specific IgG responses and identified several salivary proteins with high plasma reactivity that triggered a Th1-skewed cellular immune response, suggesting their potential role in host defense and vaccine development. At last, Borges-Fernandes et al. examined the impact of blocking MHC class I-related protein 1 (MR1) on the internalization of *L. infantum* by host phagocytic cells. Their findings indicated that MR1 blockade led to increased TNF-α and IL-10 production in VL patients, whereas asymptomatic individuals exhibited a comparatively lower cytokine response. Additionally, MR1 blockade reduced IFN-γ levels and elevated Th2-associated cytokines, underscoring a potential protective role of MR1 in modulating immune responses during VL. Finally, this Research Topic also received two submissions that, although ultimately not endorsed during peer review, nonetheless reflected the relevance of the subject matter.
